# Role of *CDH1* gene variants and E-cadherin localization in gastric mucosal cancerization

**DOI:** 10.3389/fonc.2025.1590680

**Published:** 2025-05-09

**Authors:** Yunbo Wu, Yunzhan Zhang, Yunkai Dai, Qi Luo, Shaoyang Lan, Xu Chen, Weijing Chen, Ruliu Li, Ling Hu

**Affiliations:** ^1^ Institute of Gastroenterology, Science and Technology Innovation Center, Guangzhou University of Chinese Medicine, Guangzhou, China; ^2^ Gastroenterology Department, First Affiliated Hospital of Guangzhou University of Chinese Medicine, Guangzhou, China

**Keywords:** E-caderin, CDH1, gastric cancer, SNP, *Helicobacer pylori*

## Abstract

**Background:**

This study investigates the role of *CDH1* gene single nucleotide polymorphisms (SNPs), mRNA transcription levels, and E-cadherin protein localization in Helicobacter pylori (Hp)-associated gastric diseases and their contribution to gastric mucosal carcinogenesis.

**Methods:**

Gastric mucosal samples were analyzed for histopathology (Hematoxylin-Eosin staining) and Hp detection (rapid urease test, Giemsa staining). SNPs at the *CDH1* gene rs16260 locus were identified via sequencing, mRNA levels were quantified by real-time PCR, and E-cadherin localization was assessed using Elivision™ Plus. Statistical analyses were performed with SPSS 25.0.

**Results:**

Participants were grouped by gastric mucosal pathology: normal (NOR), gastric inflammation (GI), gastric atrophy (GA), gastric premalignant lesion (GPL), severe dysplasia (GSD), and gastric cancer (GC). No significant differences were found in *CDH1* rs16260 genotypes. However, *CDH1* transcription was higher in GC compared to NOR and GPL groups. Intestinal metaplasia showed lower *CDH1* mRNA levels. E-cadherin expression was higher in GSD and GC compared to other groups. Localization analysis revealed decreased membrane-bound E-cadherin with increased cytoplasmic expression as lesion severity increased. Quantitative analysis showed higher E-cadherin expression in GA than other groups, indicating an initial rise followed by a decline in malignancy. Regression analysis suggested that elevated *CDH1* mRNA increased gastric cancer risk, while E-cadherin cytoplasmic ectopic expression heightened the risk of precancerous lesions and gastric cancer.

**Conclusion:**

The A allele of the CDH1 gene rs16260 locus show no effect in gastric mucosal pathological evolution, while the elevated mRNA transcription levels potentially increasing the risk of gastric cancer. The loss and ectopic expression of E-cadherin may be significant risk factors for malignant transformation in the gastric mucosa.

## 1 Introduction

Gastric cancer (GC) is one of the most prevalent cancers globally, ranking fifth in incidence and fourth in mortality among all cancers, posing a significant threat to human health (1). The development of GC results from the combined effect of multiple factors, including genetic predispositions, dietary habits, smoking, and alcohol consumption, with Helicobacter *pylori* (Hp) infection being a major risk factor in non-cardia gastric cancer (2). Long-term Hp infection can lead to gradual gastric mucosal atrophy and intestinal metaplasia, and without timely intervention, may ultimately progress to dysplasia and carcinogenesis (3). Diseases encompassing the pathological changes from benign to malignant in gastric mucosa are collectively termed Hp-associated gastric diseases (HpGD), including gastric ulcers, chronic gastritis, and gastric cancer.

E-cadherin is a calcium-dependent type I transmembrane glycoprotein that plays a crucial role in maintaining cell adhesion, stabilizing the cytoskeleton, and preserving the normal morphological structure of tissues and organs (4). Abnormal E-cadherin expression is closely associated with the occurrence of various cancers, including breast cancer, ovarian cancer, and colorectal cancer. Decreased or absent expression of E-cadherin is significantly correlated with the high malignancy, invasiveness, and metastasis of tumor tissues (5–7). Previous studies have also indicated abnormal expression of E-cadherin in gastric cancer tissues, closely correlated with clinical characteristics such as Lauren classification, depth of infiltration, degree of differentiation, lymph node metastasis, and TNM staging (8, 9). However, previous studies mainly focused on the expression changes of E-cadherin in gastric cancer, and the specific patterns of E-cadherin protein variation in the malignant transformation of gastric mucosa remain unclear.

*CDH1*, gene encoding E-cadherin, is located in the q22.1 region of chromosome 16, comprising 16 exons and 15 introns. Recent studies have identified multiple single nucleotide polymorphisms (SNPs) in the promoter region that regulate *CDH1* transcriptional activity, with the *CDH1*-160 (rs16260) locus being among the more common variants (10). It has been reported that the *CDH1–*160 C>A polymorphism significantly influences the occurrence, development, and prognosis of various cancers (11–13), while its specific role in gastric cancer remains controversial (14, 15).

This study aims to investigate the roles of *CDH1* gene SNPs, mRNA expression levels, and qualitative and locational expression of its protein E-cadherin in HpGD, exploring these factors longitudinally in the benign and malignant pathological evolution of gastric mucosa, providing potential early warning indicators for gastric cancer ultimately.

## 2 Methods

### 2.1 Study population and specimens collection

This study recruited a total of 504 participants from November 2016 to September 2019 from the Endoscopy Center of the First Affiliated Hospital of Guangzhou University of Chinese Medicine, including patients diagnosed with chronic gastritis, gastric ulcers, and gastric cancer, as well as participants undergoing routine health examinations. General information and clinical data were collected from all participants. Four tissue samples were collected from suspected lesions (around ulcers for ulcer patients; from the gastric antrum for health examination participants) for histopathological classification, Hp detection, *CDH1* (rs16260) gene polymorphism analysis, *CDH1* gene mRNA transcription level measurement, and qualitative and locational analysis of E-cadherin expression. This research was approved by the Ethics Committee of the First Affiliated Hospital of Guangzhou University of Chinese Medicine (Ethics Approval No [2015]: 009), and all participants provided informed consent.

### 2.2 Pathology evaluation and Hp detection

The gastric mucosal tissue was stained using the Hematoxylin and Eosin (HE) method. Pathological evaluation followed the Sydney System (16), categorizing participants into six groups based on endoscopic findings and gastric mucosal pathological features, including inflammation severity, activity, atrophy degree, intestinal metaplasia degree, dysplasia degree, and cancerous changes. Participants were classified as follows: (1) Normal group (NOR): No apparent abnormalities observed on gastric mucosa under endoscopy, with no significant pathological changes or only mild inflammation. (2) Gastric inflammation group (GI): Significant lesions observed on gastric mucosa under endoscopy, accompanied by chronic or active inflammatory changes, without atrophy, intestinal metaplasia, or dysplasia. (3) Gastric atrophy group (GA): Intrinsic glandular atrophy of gastric mucosa, with or without chronic or active inflammation, without intestinal metaplasia or dysplasia. (4) Gastric premalignant lesion group (GPL): Intestinal metaplasia present in gastric mucosa or mild to moderate dysplastic changes, with or without gastric mucosal atrophy, chronic or active inflammation. (5) Gastric severe dysplasia group (GSD): Severe dysplastic changes observed in gastric mucosa, with or without inflammation, atrophy, or intestinal metaplasia. (6) Gastric cancer group (GC): Participants meeting diagnostic criteria for gastric cancer. Hp infection was detected using rapid urease testing and the Giemsa staining method (17). A positive result in either test indicated Hp infection, classified as mild, moderate, or severe based on Giemsa staining results.

### 2.3 *CDH1* SNP detection

This study conducted single nucleotide polymorphism (SNP) testing on the *CDH1*-160 (rs16260) locus of gastric mucosal tissue. The gene sequence for this locus was retrieved from the NCBI database, and primers were designed and synthesized using Primer Expression2.0 software as follows:

Outer amplification: Upstream primer: ctgtactcccagctactagag.
Downstream primer: cgtaccgctgattggctgag.
Inner amplification: Upstream primer: cttgagcccaggagttcgag.
Downstream primer: gccacagccaatcagcag.

DNA from gastric mucosal tissue was extracted using the HiPure Tissue DNA Kits (Magen, Cat. No: D3121-02), followed by nested PCR amplification (outer and inner rounds) using TaKaRa LA Taq^®^ with GC Buffer (Takara, Cat. No: RR02AG). The amplification conditions were set as follows: 94°C for 30 seconds, 61.4°C for 30 seconds, and 72°C for 30 seconds, for a total of 35 cycles. Extension was performed at 72°C for 10 minutes, and the reaction was terminated at 10°C for 30 seconds. PCR products were separated and analyzed using 1% agarose gel electrophoresis. A clear, single band of 339 bp confirmed successful amplification. Subsequently, direct sequencing was performed on the amplified products.

### 2.4 Quantitative real-time PCR reaction

Quantitative real-time PCR (qPCR) was employed to assess the mRNA transcription levels of the *CDH1* gene. Primer sequences were 5’-ccttagaggtgggtgactac-3’ (forward) and 5’-caagaatccccagaatggcag-3’ (reverse). The procedure involved several specific steps: RNA extraction from gastric mucosal tissue using RNAiso Plus solution (TaKaRa, Cat. No: AKA1202); reverse transcription using the ReverTra Ace qPCR RT Kit (TOYOBO, Cat. No: FSQ-101); and fluorescence-based qPCR using SYBR Premix Ex TaqTM (TliRNaseH Plus) (TaKaRa, Cat. No: AK6006). Reaction conditions included an initial denaturation at 95°C for 30 seconds, followed by amplification cycles at 95°C for 5 seconds, 60°C for 30 seconds, and 72°C for 1 minute, repeated 40 times. Melting curve analysis was conducted with a denaturation step at 95°C for 15 seconds, annealing at 65°C for 1 minute, and final denaturation at 95°C for 15 seconds. The relative gene expression differences were evaluated using the 2^-ΔΔCp method, with Actinβ serving as the internal reference gene.

### 2.5 E-cadherin detection

Following dewaxing of paraffin-embedded gastric mucosal tissue, E-cadherin protein localization was detected using the Elivision™ plus labeling method. Endogenous peroxidase activity was inhibited by immersion in 3% H2O2 at room temperature (20-25°C) for 10 minutes. Subsequently, sections were incubated with the Anti-E-cadherin antibody (EP700Y) (dilution 1:1500, ab40772, Abcam) at 4°C overnight (mainly recognized the C-terminal cytoplasm domain of human E-cadherin). After washing with PBS, sections were incubated with Goat Anti-Rabbit IgG H&L (HRP) (ab6721, Abcam) as the secondary antibody for 30 min at 37°C.DAB solution was applied for color development, followed by mounting and observation under an optical microscope. The evaluation of protein expression was based on a combined of the positive cell percentage and staining intensity. Specifically, the scoring for positive cell percentage includes: 0 points: ≤25% positive cells, 1 point: 26%–75% positive cells, 2 points: ≥76% positive cells; the scoring for staining intensity includes: 0 points: No staining, 1 point: Light yellow staining, 2 points: Brown staining. The final score(ranging from to 4) was calculated as the sum of the two scores and categorized as follow: 0–1: Negative (–), 2: Weakly positive (+), 3: Positive (++), 4: Strongly positive (+++). Additionally, protein localization of E-cadherin was assessed according to staining patterns.

### 2.6 Statistical analysis

Statistical analysis was performed using SPSS 25.0 software. For categorical data such as genotype distribution and E-cadherin protein expression, Chi-square test or Fisher’s exact test was employed. Ordinal data including Hp infection severity and E-cadherin protein quantitative scores were analyzed using Kruskal-Wallis H test. Analysis of *CDH1* gene mRNA transcription levels utilized either one-way analysis of variance (ANOVA) or Kruskal-Wallis test, with Spearman’s rank correlation assessing the relationship between *CDH1* gene mRNA transcription levels and severity of gastric mucosal histopathology. Multivariate logistic regression analysis was conducted to evaluate the association between *CDH1* gene polymorphisms, *CDH1* gene mRNA transcription levels, E-cadherin protein qualitative localization expression, and gastric mucosal histopathological features, using odds ratios (OR) and 95% confidence intervals (CI). Power analysis was performed to examine the statistical power. The significance level was set at α=0.05, indicating P<0.05 was considered statistically significant.

## 3 Results

### 3.1 Basic characteristics

**Figure 1:**
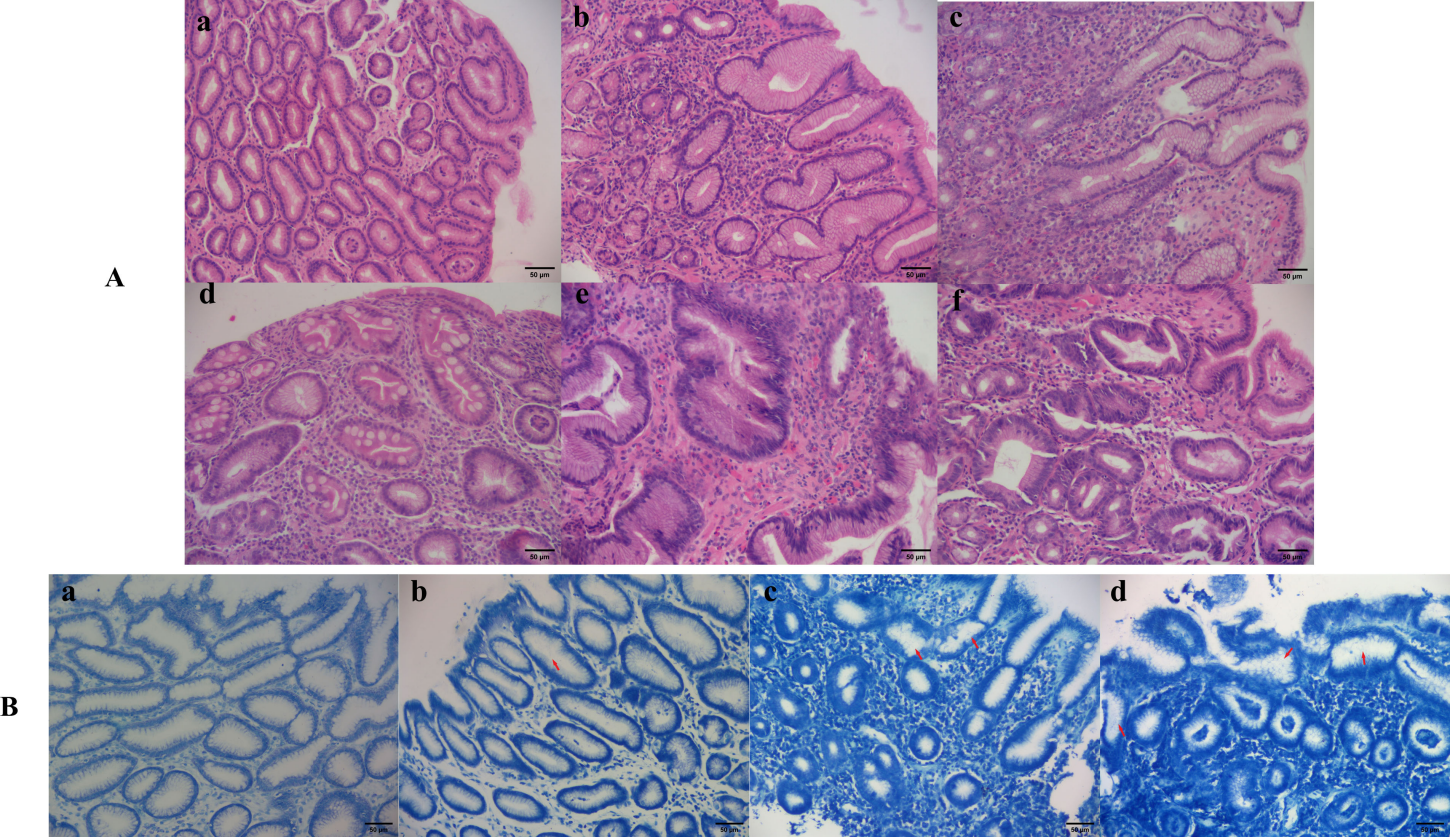
Different histopathological features and Hp infection states of gastric mucosa. **(A)** Histopathological features of gastric mucosa (hematoxylin-eosin staining 200×): (a) Normal gastric mucosa; (b) Gastric mucosa with moderate inflammation; (c) Gastric mucosa with moderate atrophy and severe inflammation; (d) Gastric mucosa with severe inflammation, severe intestinal metaplasia and mild dysplasia; (e) Moderate atrophic gastric mucosa with moderate dysplasia and severe inflammation; (f) Cancerous gastric mucosa. **(B)** Hp infection situations (methylene blue staining 200×): (a) Negative; (b) Mild; (c) Moderate; (d) Severe.

This study included a total of 504 participants, comprising 270 males and 234 females, with a mean age of 49.74 ± 11.98 years and an average disease duration of 1315.61 ± 1903.71 days. The overall Hp infection rate was 75.6%. Baseline characteristics of each pathological group are presented in **Table 1**. There were no statistically significant differences in gender distribution and age among the groups. However, there was a statistically significant difference in Hp infection rates among the groups (*P* = 0.001). Specifically, the Hp infection rate in the GPL group was higher than that in the NOR group (80.8% vs 52.9%, P = 0.004) and the GI group (80.8% vs 64.3%, *P* = 0.020). **Figure 1** illustrates the relationship between different Hp infection statuses and histopathological features of gastric mucosa in the participants.

**Table 1:** Demographic information of study subjects.

Pathological groups	NOR (%) (n=34)	GI (%) (n=98)	GA (%) (n=47)	GPL (%) (n=233)	GSD (%) (n=64)	GC (%) (n=24)	*P* ^a,b^
Age (mean ± SD)	49.50 ± 11.99	48.15 ± 12.66	48.24 ± 10.81	50.52 ± 48.24	50.62 ± 12.29	53.08 ± 15.92	0.411^a^
Gender (male)	16(47.1%)	49(50.0%)	27(55.1%)	124(53.0%)	37(57.8%)	17(68.0%)	0.591^b^
H. Py*lori* (+)	18(52.9%)	63(64.3%)	39(79.6%)	189(80.8%)	51(79.7%)	21(84.0%)	**0.001^b^**

NOR, Normal control group; GI, Gastric inflammation group; GA, Gastric atrophy group; GPL, Gastric precancerous lesions group; GSD, Gastric severe dysplasia group; GC, Gastric cancer group. ^a^p-value was calculated by One-way analysis of variance (ANOVA). ^b^p-value was calculated by chi-square test.

The bold values indicates that the Hp infection rates in different pathological groups were significantly different (P=0.001<0.05). Specifically, the Hp infection rate in the GPL group was higher than that in the NOR group (80.8%vs52.9%, P=0.004) and the GI group (80.8%vs 64.3%, P= 0.020).

### 3.2 *CDH1* SNP and HpGD

**Figure 2:**
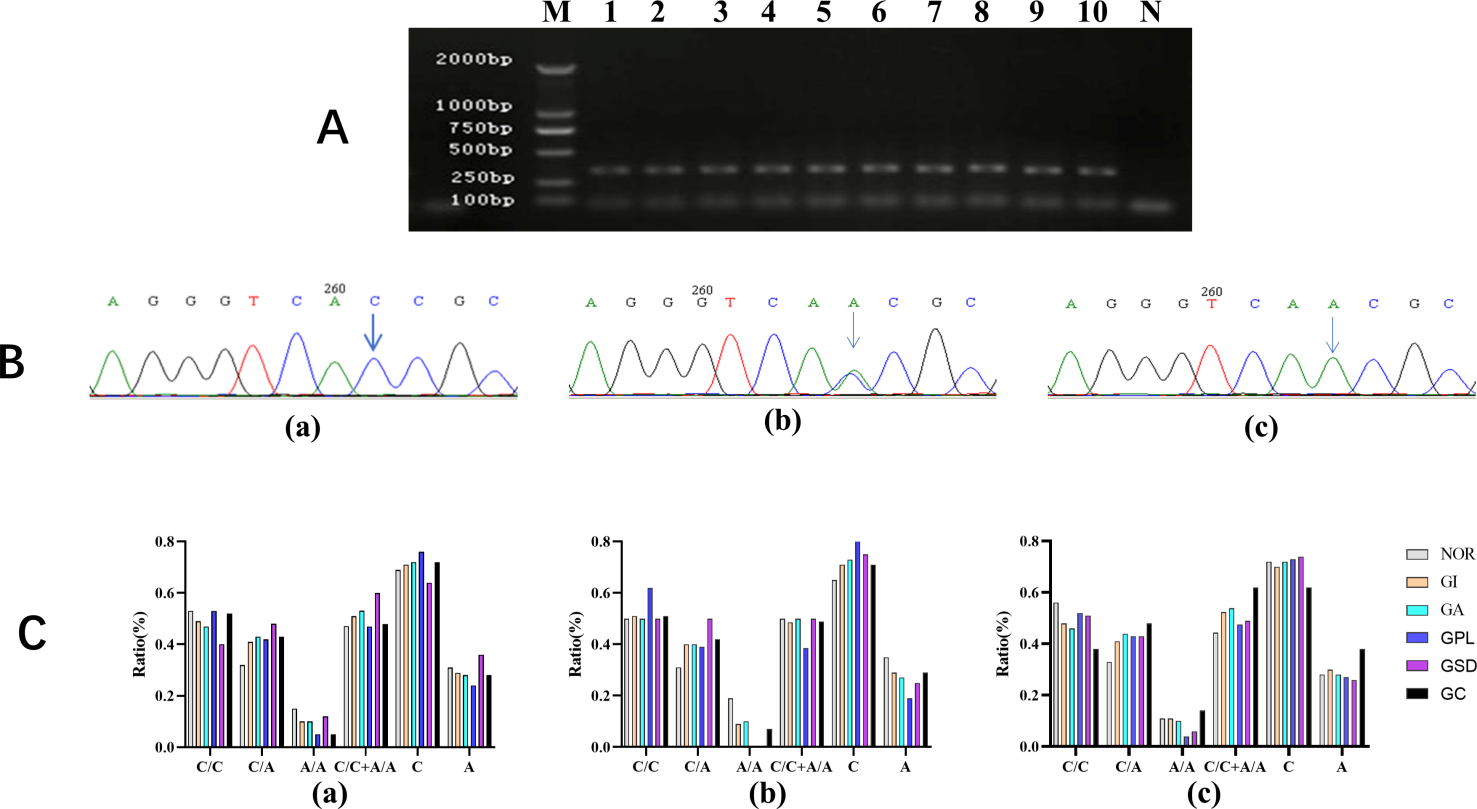
Genotyping for *CDH1–*160 SNP and its frequency in pathological groups. **(A)** gel electrophoresis of PCR product, M: Marker; Lane 1 to 10 represents 10 different individuals. **(B)** Results of genotyping: (a) CC homozygous wild, (b) CA heterozygous mutant, (c) GG homozygous mutant type. **(C)** Distribution of genotype in pathological groups: (a) all subjects, (b) *H. pylori*-negative subjects. (c) *H. pylori*-positive subjects.

The PCR product of *CDH1* is displayed in **Figure 2A**, and the SNP at the *CDH1*-160 (rs16260) locus was identified as a C>A mutation, presenting three genotypes: C/C wild-type homozygote, C/A mutant heterozygote, and A/A mutant homozygote (**Figure 2B**). Hardy-Weinberg equilibrium testing indicated that the genetic balance of this locus in the subjects was maintained (P=0.87>0.05), suggesting they originated from the same Mendelian population. The distribution of genotypes across different pathological groups is shown in **Figure 2C**(a), with no statistically significant differences in genotype or allele distribution among the groups (genotype *P*=0.465, allele *P*=0.863). To exclude the impact of Hp infection on gastric mucosal pathology, statistical analysis was performed separately for Hp-negative (**Figure 2C**(b)) and Hp-positive subjects (**Figure 2C**(c)). The results indicated no significant differences in *CDH1*-160 (rs16260) genotype or allele distribution among the pathological groups, regardless of Hp infection status (genotype: Hp-positive *P*=0.938, Hp-negative *P*=0.642; allele: Hp-positive P=0.990, Hp-negative *P*=0.825). To further investigate the effect of the *CDH1–*160 SNP on gastric mucosal pathology, the pathological changes in the gastric mucosa of different genotype carriers were analyzed. As shown in **Table 2**, there were no statistically significant differences in the likelihood of atrophy, intestinal metaplasia, or dysplasia in the gastric mucosa among patients with different genotypes, regardless of Hp infection status.

**Table 2:** *CDH1–*160 SNPs and gastric pathological change.

Gastric mucosa lesions	C/C (%) (N=255)	C/A (%) (N=211)	A/A (%) (N=37)	χ^2^	*P*	C/C (%) (N=255)	C/A+A/A (%) (N=249)	χ^2^	*P*
H. *pylori* negative									
GA (+)	18 (28.1)	17 (35.4)	3 (30.0)	0.687	0.765	18 (28.1)	20 (34.5)	0.573	0.557
IM (+)	20 (31.3)	10 (20.4)	2 (20.0)	1.900	0.394	20 (31.3)	12 (20.3)	1.899	0.218
DYS (+)	31 (48.4)	25 (51.0)	3 (30.0)	1.482	0.477	31 (48.4)	28 (47.5)	0.012	1.000
H. *pylori* positive									
GA (+)	113 (59.2)	109 (66.9)	14 (51.9)	3.472	0.186	113 (59.2)	123 (65.7)	1.256	0.292
IM (+)	59 (30.9)	64 ( (39.3)	5 (18.5)	5.725	0.061	59 (30.9)	69 (36.3)	1.257	0.279
DYS (+)	128 (67.0)	104 (63.8)	14 (51.9)	2.45	0.309	128 (67.0)	118 (62.1)	1.004	0.336

GA, Gastric atrophy; IM, Intestinal metaplasia; DYS, Dysplasia.

### 3.3 *CDH1* mRNA and HpGD

**Figure 3:**
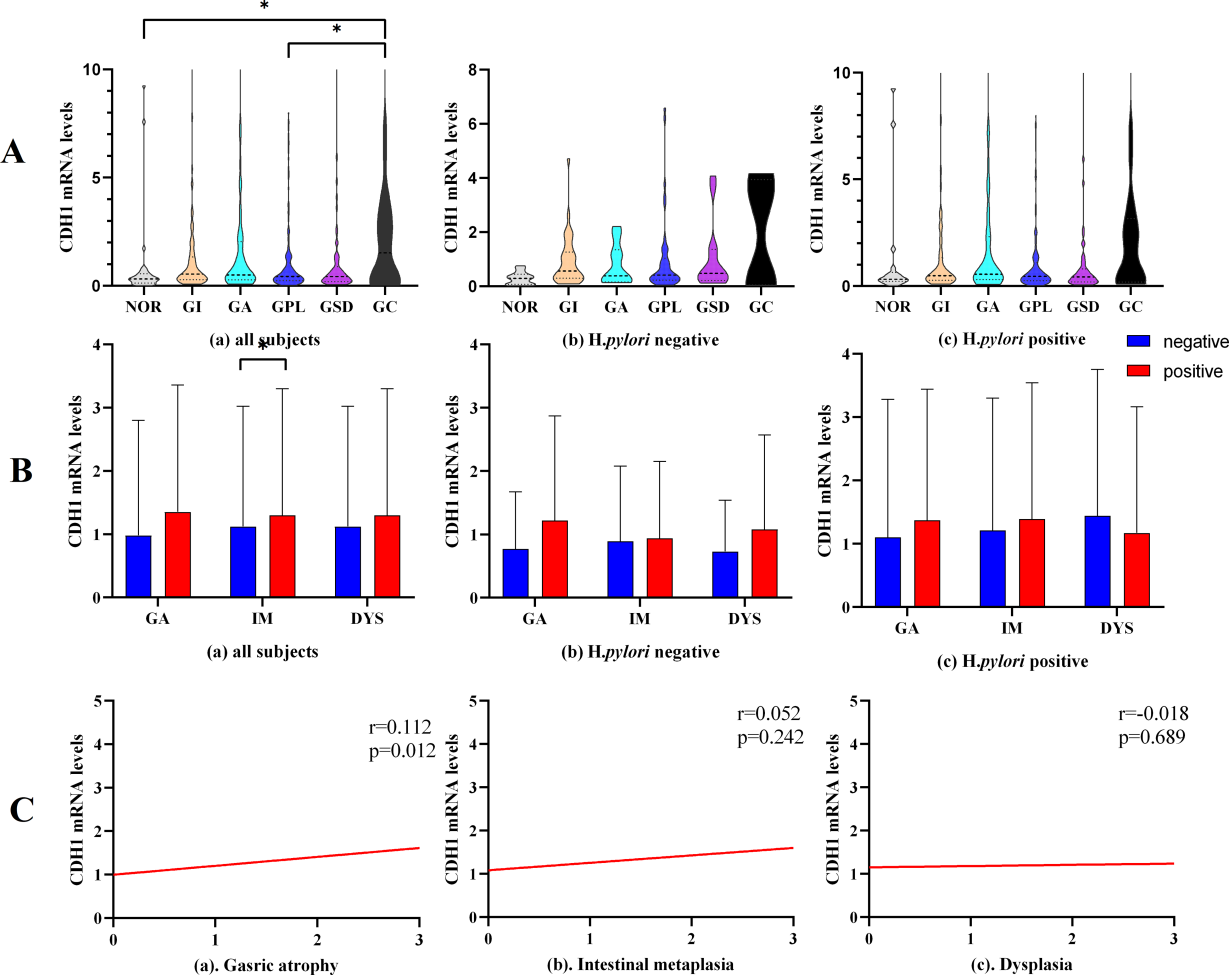
*CDH1* mRNA expression. **(A)** *CDH1* mRNA expression in different pathological groups. **(B)** *CDH1* mRNA expression in gastric mucosa with different pathological changes. **(C)** Correlation analysis between *CDH1* mRNA level and gastric pathological change. GA, gastric atrophy, IM, intestinal metaplasia, DYS, dysplasia. *P<0.05.

The expression levels of mRNA in different pathological groups are illustrated in **Figure 3A**. The non-parametric tests revealed statistically significant differences in mRNA expression levels among the groups; specifically, the mRNA transcription level in the GC group was higher than that in the NOR group (2.34 vs. 0.83, *P*=0.046) and the GPL group (2.34 vs. 1.06, *P*=0.024). Further analysis of the relationship between *CDH1* mRNA transcription levels and gastric mucosal pathology indicated that overall, the mRNA transcription level of the *CDH1* gene was significantly lower in intestinal gastric mucosal tissue compared to non-intestinal tissue (1.35 vs. 0.97, *P*=0.030, **Figure 3B**(a)). Correlation analysis showed a positive correlation between the degree of gastric mucosal atrophy and *CDH1* gene mRNA expression (r=0.112, *P*=0.012, **Figure 3C**(a)).

### 3.4 E-cadherin expression and HpGD

**Figure 4:**
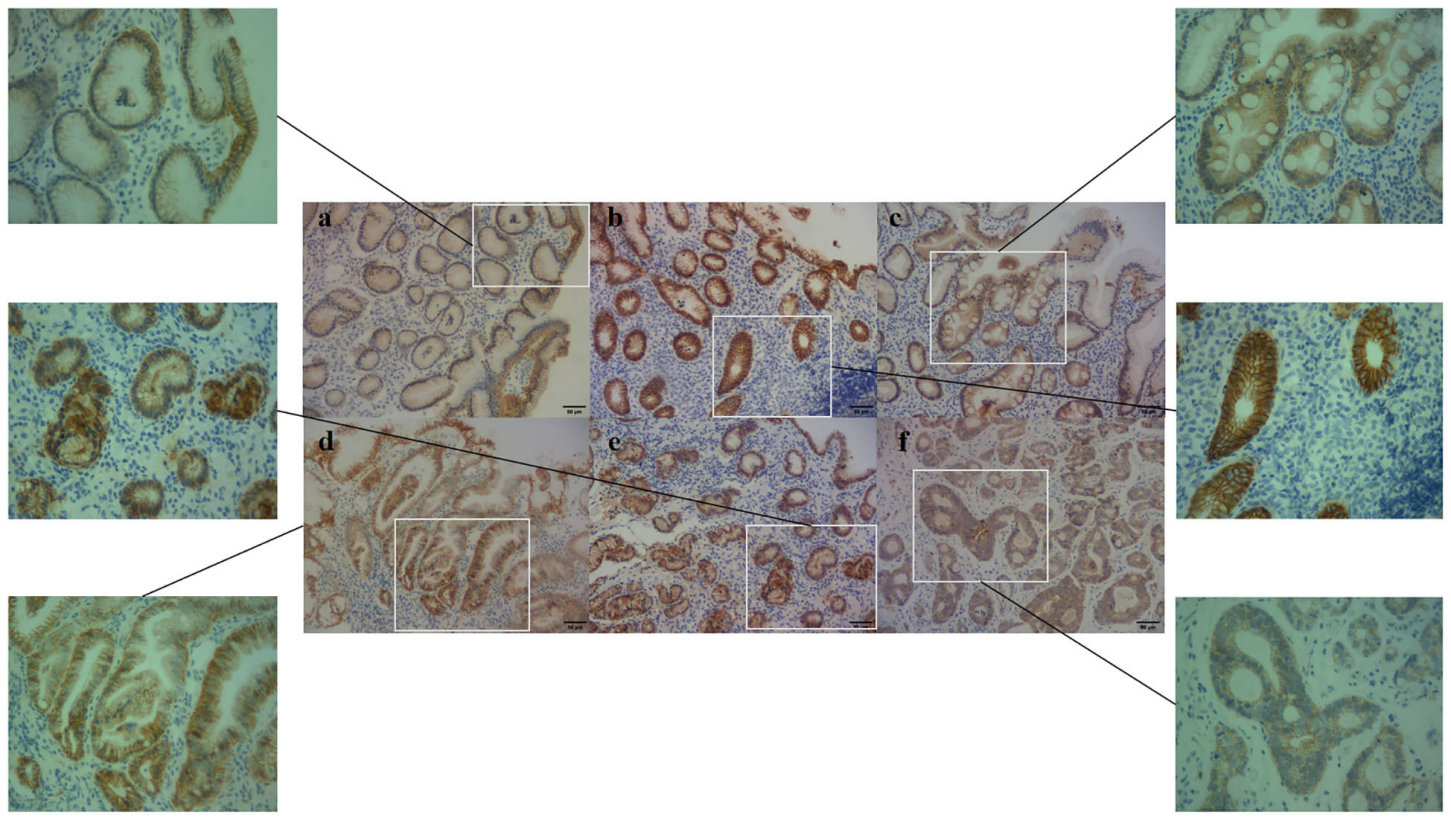
E-cadherin expression in different pathological groups (200× and 400x). **(a)** NOR group, E-cadherin membrane expression, **(b)** GPL group, E-cadherin both membrane and cytoplasm expression, **(c)** GPL group, E-cadherin cytoplasm expression, **(d)** GSD group, E-cadherin membrane expression, **(e)** GSD group, E-cadherin cytoplasm expression, **(f)** GC group, E-cadherin cytoplasm expression.

**Figure 5:**
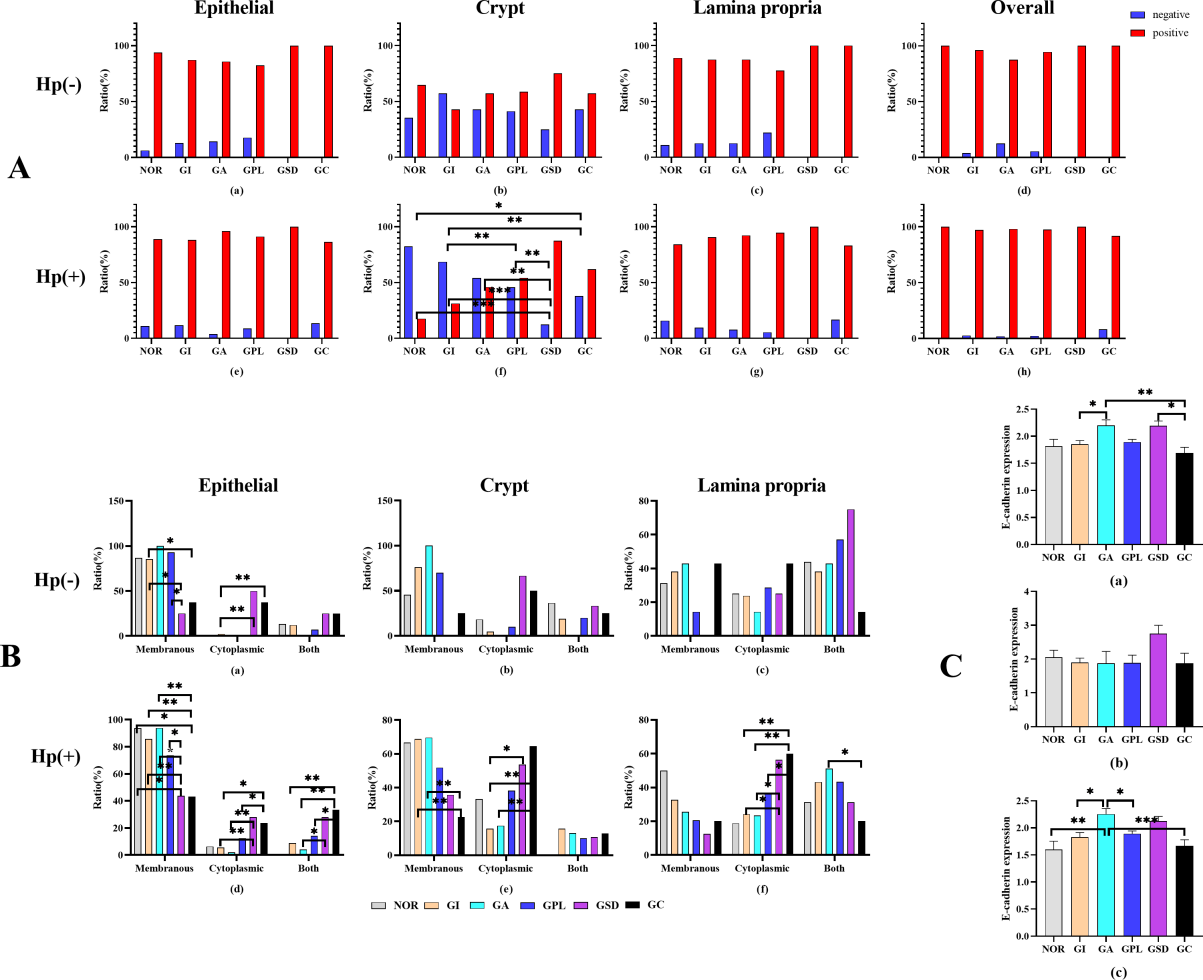
**(A)** Ratio of E-cadherin in different area of histopathological groups. **(B)** E-cadherin situation in histopathological groups. **(C)** Semiquantitative analysis in histopathological groups. (a) all subjects, (b) *H. pylori*-negative subjects. (c) *H. pylori*-positive subjects. *P<0.05, **P<0.01.

E-cadherin protein, an important adhesion molecule located primarily on the membrane of epithelial cells, exhibited varying expression across different pathological groups, as shown in **Figure 4**. Analysis of E-cadherin expression in different regions of the gastric mucosa (**Figure 5A**) revealed higher expression rates in the GSD group within the gastric pits of Hp-positive subjects compared to the NOR, GI, GA, and GPL groups (87.5% vs. 17.6%, 54.2%, 31.4%, 46.0%, *P=* 0.001, <0.001, 0.002, 0.006, respectively). The GC group also showed higher expression rates than the NOR and GI groups (62.0% vs. 17.6%, 21.4%, *P*= 0.024, 0.005), while the GPL group had higher expression rates than the GI group (54.2% vs. 31.4%, *P*=0.002). However, no significant differences were observed in the epithelial area, lamina propria, or overall E-cadherin expression rates.

Subsequent analysis of the localization of E-cadherin protein expression revealed significant differences among the pathological groups (**Figure 5B**). In Hp-negative subjects, the epithelial region showed higher membrane expression rates in the GI group compared to the GSD and GC groups (*P*=0.040 and *P*=0.030, respectively), and the GPL group had higher membrane expression rates than the GSD group (*P*=0.040). Conversely, the GI group had lower cytoplasmic expression rates than the GSD and GC groups (*P*=0.001 and *P*=0.003, respectively). In Hp-positive subjects, the membrane expression rates in the NOR, INF, GA, and GPL groups were higher than in the GSD (*P*=0.013, <0.001, <0.001, and 0.011, respectively) and GC groups (*P*=0.006, <0.001, <0.001, and 0.001, respectively), with the GA group exhibiting higher membrane expression rates than the GPL group (*P*=0.036). Additionally, the cytoplasmic expression rates in the GSD and GC groups were higher than in the GI (*P*=0.08 and 0.022) and GA groups (*P*=0.007 and 0.021), with the GC group showing higher membrane and cytoplasmic co-expression rates compared to the GI, GA, and GPL groups (*P*=0.002, 0.002, and 0.015, respectively). The GSD group also had higher membrane and cytoplasmic co-expression rates than the GA group (*P*=0.020). In the gastric pits, the membrane expression rates in the GI and GA groups were higher than in the GC group (*P*=0.004 and 0.008), while the cytoplasmic expression rates in the GSD and GC groups were higher than in the GI group (*P*=0.028 and 0.001) and the GC group had higher cytoplasmic expression rates than the GA group (*P*=0.009). In the lamina propria, the cytoplasmic expression rates in the GSD and GC groups were higher than in the GI (*P*=0.012 and 0.001) and GA groups (*P*=0.044 and 0.005), with the GC group showing higher cytoplasmic expression rates than the GPL group (*P*=0.049). The GA group exhibited higher membrane and cytoplasmic co-expression rates than the GC group (*P*=0.029). Overall, as the malignancy of the gastric mucosa progressed, E-cadherin protein expression shifted from membrane expression to combined membrane and cytoplasmic expression and then predominantly to cytoplasmic expression.

Finally, we investigated the E-cadherin protein expression levels among different pathological groups. The results (**Figure 5C**) indicated significant differences in E-cadherin protein expression, with higher levels in the GA and GSD compared to the GC group (*P*=0.041 and 0.004) and higher levels in the GA compared to the GI group (*P*=0.028). In Hp-positive subjects, the GA group showed higher E-cadherin protein expression levels than the NOR, GI, GPL, and GC groups (*P*=0.009, 0.011, 0.031, and 0.001, respectively), whereas no significant differences were observed among Hp-negative subjects (*P*=0.576). These preliminary findings suggest that Hp infection may influence E-cadherin protein expression to some extent.

### 3.5 Multivariant logistic regression analysis

To further investigate the effects of the *CDH1* gene (rs16260) SNP, *CDH1* mRNA transcription levels, and E-cadherin protein localization on the benign and malignant pathological evolution of the gastric mucosa, a multivariate logistic regression analysis was performed. The results indicated that overexpression of *CDH1* mRNA significantly increased the risk of developing GC (OR=1.37, 95% CI: 1.01-1.85). Aberrant expression of E-cadherin protein significantly increased the risk of developing GPL, GSD, and GC. Specifically, in the epithelial region, E-cadherin cytoplasmic ectopic expression increased the risk of GSD and GC by 20.53 (OR=20.53, 95% CI: 2.41-174.70) and 16.80 times (OR=16.80, 95% CI: 2.07-136.49), respectively. Membrane and cytoplasmic co-expression increased the risk of GSD and GC by 9.33 (OR=9.33, 95% CI: 1.81-48.24) and 10.64 times (OR=10.64, 95% CI: 2.25-50.31), respectively. In the crypt region, E-cadherin cytoplasmic ectopic expression significantly increased the risk of developing GC (OR=6.42, 95% CI: 1.33-31.03). In the lamina propria, E-cadherin cytoplasmic ectopic expression increased the risk of developing GPL, GSD, and GC by 1.27 (OR=1.27, 95% CI: 1.21-8.84), 1.82 (OR=1.82, 95% CI: 2.14-36.37), and 1.64 times (OR=1.64, 95% CI: 1.49-14.47), respectively (**Table 3**).

**Table 3:** Multivariable logistic regression analysis.

Multivariate logistic regression analysis	GI vs NOR		GA vs NOR		GPL vs NOR		GSD vs NOR		GC vs NOR	
	OR (95% CI)	*P*	OR (95% CI)	*P*	OR (95% CI)	*P*	OR (95% CI)	*P*	OR (95% CI)	*P*
*CDH1–*160 genotype										
C/C	Ref	–	Ref	–	Ref	–	Ref	–	Ref	–
C/A	1.33(0.56, 3.14)	0.52	1.41(0.54, 3.69)	0.48	1.33(0.60, 2.96)	0.48	1.28(0.52, 3.16)	0.60	1.75(0.56, 5.47)	0.34
A/A	1.72(0.22, 2.42)	0.60	1.73(0.18, 2.95)	0.66	1.32(0.10, 1.02)	0.05	1.31(0.07, 1.45)	0.14	1.97(0.19, 5.02)	0.97
C/C+A/A	1.17 (0.54,2.56)	0.69	1.27 (0.53,3.06)	0.59	1.03 (0.50,2.12)	0.93	0.99 (0.43,2.28)	0.99	1.69 (0.59,4.80)	0.33
*CDH1* mRNA	1.16(0.86, 1.56)	0.32	1.26(0.93, 1.70)	0.14	1.12(0.84, 1.49)	0.44	1.12(0.82, 1.54)	0.46	**1.37(1.01, 1.85)**	**0.04**
E-cadherin expression	0.99(0.54, 1.79)	0.97	1.04(0.53, 2.02)	0.92	0.80(0.46, 1.39)	0.42	0.78(0.41, 1.49)	0.45	1.26(0.58, 2.73)	0.56
Epithelial location										
Membrane	Ref	–	Ref	–	Ref	–	Ref	–	Ref	–
Cytoplasmic	1.49(0.17, 12.85)	0.72	0.54(0.03, 8.94)	0.67	4.29(0.56, 33.10)	0.16	**20.53(2.41, 174.70)**	**0.01**	**16.80(2.07, 136.49)**	**0.01**
Both	1.61(0.34, 7.55)	0.55	0.54(0.07, 4.03)	0.55	2.52(0.57, 11.20)	0.23	**9.33(1.81, 48.24)**	**0.01**	**10.64(2.25, 50.31)**	**<0.01**
Crypt location										
Membrane	Ref	–	Ref	–	Ref	–	Ref	–	Ref	–
Cytoplasmic	0.37(0.07, 1.83)	0.22	0.47(0.08, 2.62)	0.39	1.57(0.38, 6.40)	0.53	3.97(0.83, 18.91)	0.08	**6.42(1.33, 31.03)**	**0.02**
Both	0.41(0.10, 1.73)	0.23	0.26(0.05, 1.48)	0.13	0.35(0.09, 1.39)	0.14	0.70(0.13, 3.79)	0.68	1.09(0.21, 5.76)	0.92
**Lamina propria location**										
Membrane	Ref	–	Ref	–	Ref	–	Ref	–	Ref	–
Cytoplasmic	1.30(0.47, 3.62)	0.61	1.49(0.45, 4.89)	0.52	**1.27(1.21, 8.84)**	**0.02**	**1.82(2.14, 36.37)**	**<0.01**	**1.64(1.49, 14.47)**	**0.01**
Both	1.31(0.55, 3.15)	0.54	1.95(0.71, 5.34)	0.19	1.37(1.00, 5.64)	0.05	1.52(0.90, 13.83)	0.07	1.90(0.29, 2.85)	0.86

NOR, Normal control group; CI, Chronic inflammation group; GA, Gastric atrophy group; GPL, Gastric precancerous lesions group; GC, Gastric cancer group.

The bold values indicates that the results of multivariant logistic regression analysis were statistically significant. Specifically, the over-expression of CDH1 mRNA significantly increased the risk of developing GC (OR=1.37, 95%CI: 1.01-1.85); the E-cadherin cytoplasmic ectopic expression in epithelial region increased the risk of GSD (OR=20.53,95%CI:2.41-174.70) and GC (OR=16.80,95% CI:2.07-136.49); membrane and cytoplasmic co-expression increased the risk of GSD (OR=9.33, 95%CI: 1.81-48.24) and GC (OR=10.64, 95%CI: 2.25-50.31). In the crypt region, E-cadherin cytoplasmic ectopic expression increased the risk of GC (OR=6.42,95%CI:1.33-31.03). In the lamina propria, E-cadherin cytoplasmic ectopic expression increased the risk of GPL (OR=1.27, 95%CI: 1.21-8.84), GSD (OR=1.82,95%CI:2.14-36.37) and GC(OR=1.64,95%CI: 1.49-14.47). All above P values were less than 0.05.

### 3.6 Statistical power analysis

To examine the statistical power of the study, *post-hoc* power analysis was conducted (**Supplementary File 2**). *Post-hoc* power analysis revealed that among the 18 statistically significant results, 12 exhibited strong power (power >80%), 6 had moderate power (power 60-80%). This indicates that our findings were supported by sufficient statistical power to detect clinically meaningful effects.

## 4 Discussion

This study investigated the impact of *CDH1*-160C>A gene (rs16260) polymorphism, *CDH1* gene mRNA transcription levels, and the differential qualitative localization of its protein, E-cadherin, on gastric mucosal pathology progression. Power analysis showed sufficient statistical power to detect clinically meaningful effects.

The *CDH1*-160C>A (rs16260) polymorphism, located in the promoter region of the gene, can influence gene expression levels and has been associated with various cancers, including breast, bladder, and ovarian cancer (11–13). Previous studies on the association between *CDH1*-160C>A polymorphism and gastric cancer have yielded conflicting results. Al-Moundhri et al. found that the *CDH1–*160 A/A genotype increased the risk of gastric cancer in the Omani population (14), whereas Menbari et al. reported no significant association between this polymorphism and gastric cancer risk in the Kurdish population (15). A meta-analysis involving 6,399 subjects suggested that the *CDH1–*160 A allele might serve as a protective factor against gastric cancer in Asian populations (OR=0.67), but no such association was found in Caucasian populations (18). Our study indicated no statistically significant differences in genotype distribution among different pathological groups, regardless of Hp infection status, and no GC related risk was found on this SNP in Chinese. This disparity may be attributed to differences in genetic background, as populations from various ethnic or geographic regions may exhibit different genetic predispositions affecting the correlation between *CDH1*-160C>A polymorphism and gastric cancer risk. Additionally, environmental and lifestyle factors, including dietary habits, smoking, alcohol consumption, and other lifestyle elements, may influence gastric cancer risk and potentially confound or obscure the relationship between *CDH1*-160C>A polymorphism and gastric cancer susceptibility.

mRNA transcription serves as a critical bridge between genes and proteins, directly influencing protein synthesis and playing a significant role in the progression and prognosis of various cancers. Our results demonstrated significant differences in *CDH1* mRNA transcription levels among different pathological groups, with notably higher levels in the GC group compared to the NOR and GPL groups. Overexpression of *CDH1* mRNA was associated with an increased risk of developing GC, which contrasts with the findings of Rossi et al., who reported significantly lower *CDH1* transcription levels in gastric cancer tissues compared to normal tissues (19). However, some studies have suggested that compensatory upregulation of *CDH1* gene expression may occur in the early stages of certain cancers (20), potentially as an adaptive response by cancer cells to maintain a degree of cell adhesion, specific to the tumor microenvironment or cancer progression stage. Our study also found lower *CDH1* transcription levels in intestinal gastric mucosal tissues compared to non-intestinal tissues. Loss of E-cadherin protein expression is a hallmark of epithelial-mesenchymal transition (EMT) (21), a process wherein cells lose adhesion properties and acquire increased invasiveness and metastatic potential. The alterations in gastric mucosal epithelium during intestinal metaplasia may modify the cellular environment, promoting EMT formation and contributing synergistically to gastric cancer development.

As a tumor suppressor, E-cadherin can inhibit cancer cell proliferation and metastasis through various pathways, and its loss is associated with the progression of multiple cancers (22). Our findings showed that in the presence of Hp infection, the expression rate of E-cadherin protein in the gastric pits of severe dysplasia and cancerous gastric mucosa was higher than in other pathological tissues. As the severity of gastric mucosal lesions increased, the expression rate of E-cadherin protein also rose, with no significant changes observed in the epithelial and glandular regions of the lamina propria. The gastric pits represent a transitional zone from the mucus neck region to the epithelial surface, containing a high density of stem cells responsible for repairing damaged gastric mucosa (23). Due to the high cellular activity and weak adhesion in this area, E-cadherin protein expression is typically low. Murata et al. found that CagA, a factor released by Hp, can interact with E-cadherin protein, disrupting the E-cadherin/β-catenin complex, leading to β-catenin activation and transformation of epithelial cells from a gastric to an intestinal phenotype (24). Consequently, we hypothesize that Hp infection damages the gastric mucosa, prompting a proliferative response in stem cells within the gastric pits to repair the damage. Concurrently, Hp’s virulence factors may impair E-cadherin function, resulting in compensatory upregulation of E-cadherin protein expression. This dual influence of Hp infection and loss of E-cadherin adhesion function significantly increases the risk of intestinal metaplasia, dysplasia, and carcinogenesis in the gastric mucosa.

Many researchers have focused solely on the differences in E-cadherin protein expression between gastric cancer tissues and normal tissues, concluding that the loss of E-cadherin protein is closely associated with the occurrence, classification, differentiation, metastasis, and prognosis of gastric cancer (9, 25). Our study, however, revealed that during the benign-to-malignant pathological progression of the gastric mucosa, E-cadherin protein expression exhibits an initial increase followed by a decrease. Specifically, E-cadherin protein expression increases from normal gastric mucosa to atrophic and dysplastic stages, but decreases once the pathology advances to the cancerous stage. The progression of gastric mucosal carcinogenesis involves the loss of contact inhibition, uncontrolled proliferation, loss of cell polarity, reduced or absent adhesion function, and increased invasiveness (9, 26). As an epithelial adhesion molecule, E-cadherin mediates intercellular adhesion, inhibiting abnormal proliferation and metastasis (27).

Moreover, E-cadherin regulates multiple signaling pathways, such as promoting the expression of p27 to inhibit cdk-cyclin activity, upregulating the Egr1 gene, promoting PTEN transcription, and inhibiting the PI3K/Akt signaling pathway, thereby suppressing malignant proliferation and differentiation of cells (28, 29). Through the Hippo pathway and RTK/SRC family kinase signaling pathways, E-cadherin mediates contact inhibition, preventing tumor cell proliferation and metastasis (30, 31). We speculate that in the early stages of gastric mucosal lesions, E-cadherin protein expression may be upregulated to exert its tumor-suppressive function compensatively. However, as the disease progresses and the severity of Hp infection and cellular malignancy increases, the expression of E-cadherin protein gradually decreases, potentially contributing to the loss of normal physiological function in cells.

Mature E-cadherin protein is located on the cell membrane, spanning inside and outside the cell, and comprises three parts: the intracellular domain, the transmembrane region, and the extracellular domain (32). The intracellular domain connects with the actin cytoskeleton by binding to various catenin (α, β, P120), mediating multiple signaling pathways that regulate cell growth and differentiation. The extracellular domain facilitates intercellular adhesion and maintains epithelial integrity (33, 34). Our findings indicated that as the pathological severity of the gastric mucosa increased, E-cadherin protein expression on the cell membrane in the epithelial, gastric pit, and lamina propria regions gradually decreased, while its cytoplasmic expression increased. Regression analysis demonstrated that the E-cadherin ectopic expression from the membrane to the cytoplasm significantly increased the risk of premalignant lesions and cancer in the gastric mucosa. Coupled with the finding that E-cadherin expression does not decrease but rather increases in the early stages of gastric mucosal lesions, we hypothesize that the E-cadherin ectopic expression might be a crucial factor in early gastric carcinogenesis.

However, several limitations also exist. Firstly, the sample sizes in different pathological groups were unbalanced, and were relatively small such GC Group (N=24), which may lower the statistical power. Although we conducted the power analysis to minimize the bias, a large cohort’s replication was still needed in the future.

## 5 Conclusion

The A allele of the *CDH1* gene rs16260 locus show no effect in gastric mucosal pathological evolution, while the elevated mRNA transcription levels potentially increasing the risk of gastric cancer. The loss and ectopic expression of E-cadherin may be significant risk factors for malignant transformation in the gastric mucosa.

## Data Availability

All relevant data is contained within the article. The original contributions presented in the study are included in the article/**Supplementary Material**, further inquiries can be directed to the corresponding author.
