# Supplementary figures and images for: Role of CDH1 gene variants and E-cadherin localization in gastric mucosal cancerization
Source: Front Oncol. 2025 May 9;15:1590680. doi: 10.3389/fonc.2025.1590680 (PMC12098069; doi:10.3389/fonc.2025.1590680)

CDH-1 (rs16260)

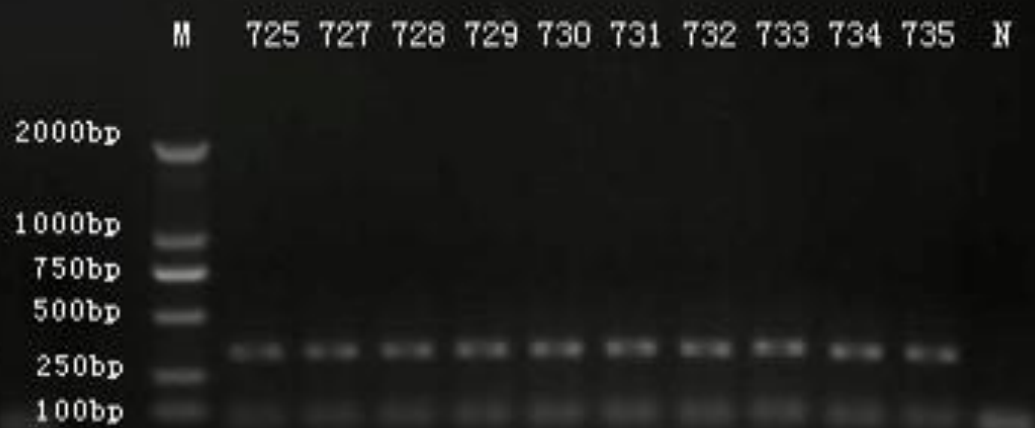

Supplement: Supplementary file 1 [file DataSheet1.pdf]
