# Supplementary material for: Role of CDH1 gene variants and E-cadherin localization in gastric mucosal cancerization
Source: Front Oncol. 2025 May 9;15:1590680. doi: 10.3389/fonc.2025.1590680 (PMC12098069; doi:10.3389/fonc.2025.1590680)
Supplement: Supplementary file 2 [file DataSheet2.docx]

**Supplementary file 2. Post-hoc power analysis**

| Category | Comparison | Cohen's h | Power | Levels of power |
| --- | --- | --- | --- | --- |
| mRNA comparison | NOR vs GC | 0.65 | 72% | Moderate |
|  | GPL vs GC | 0.78 | 89% | Strong |
| E-cadherin comparison | GSD vs NOR | 2.03 | 99% | Strong |
|  | GSD vs GI | 1.28 | 93% | Strong |
|  | GSD vs GA | 1.79 | 96% | Strong |
|  | GSD vs GPL | 1.43 | 97% | Strong |
|  | GC vs NOR | 1.92 | 88% | Strong |
|  | GC vs GI | 0.37 | 65% | Moderate |
|  | GPL vs GI | 0.45 | 78% | Moderate |
| Regression analysis |  |  |  |  |
| mRNA expression | GC vs NOR | - | 68% | Moderate |
| Epithelial location (Cytoplasmic) | GSD vs NOR | - | 99% | Strong |
|  | GC vs NOR | - | 99% | Strong |
| Epithelial location (both) | GSD vs NOR | - | 98% | Strong |
|  | GC vs NOR | - | 99% | Strong |
| Crypt location (Crypt location) | GC vs NOR | - | 85% | Strong |
| Lamina propria location (Cytoplasmic) | GPL vs NOR | - | 73% | Moderate |
|  | GSD vs NOR | - | 81% | Strong |
|  | GC vs NOR | - | 79% | Moderate |
